# Hyperspectral Imaging Using Intracellular Spies: Quantitative Real-Time Measurement of Intracellular Parameters *In Vivo* during Interaction of the Pathogenic Fungus *Aspergillus fumigatus* with Human Monocytes

**DOI:** 10.1371/journal.pone.0163505

**Published:** 2016-10-11

**Authors:** Sara Mohebbi, Florian Erfurth, Philipp Hennersdorf, Axel A. Brakhage, Hans Peter Saluz

**Affiliations:** 1 Department of Cell and Molecular Biology, Leibniz Institute for Natural Product Research and Infection Biology – Hans Knöll Institute, Jena, Germany; 2 Department of Photonics and Sensorics of Jena, Felsbachstrasse, Jena, Germany; 3 Department of Molecular and Applied Microbiology, Leibniz Institute for Natural Product Research and Infection Biology – Hans Knöll Institute, Jena, Germany; 4 Friedrich Schiller University, Jena, Germany; Universidade de Sao Paulo, BRAZIL

## Abstract

Hyperspectral imaging (HSI) is a technique based on the combination of classical spectroscopy and conventional digital image processing. It is also well suited for the biological assays and quantitative real-time analysis since it provides spectral and spatial data of samples. The method grants detailed information about a sample by recording the entire spectrum in each pixel of the whole image. We applied HSI to quantify the constituent pH variation in a single infected apoptotic monocyte as a model system. Previously, we showed that the human-pathogenic fungus *Aspergillus fumigatus* conidia interfere with the acidification of phagolysosomes. Here, we extended this finding to monocytes and gained a more detailed analysis of this process. Our data indicate that melanised *A*. *fumigatus* conidia have the ability to interfere with apoptosis in human monocytes as they enable the apoptotic cell to recover from mitochondrial acidification and to continue with the cell cycle. We also showed that this ability of *A*. *fumigatus* is dependent on the presence of melanin, since a non-pigmented mutant did not stop the progression of apoptosis and consequently, the cell did not recover from the acidic pH. By conducting the current research based on the HSI, we could measure the intracellular pH in an apoptotic infected human monocyte and show the pattern of pH variation during 35 h of measurements. As a conclusion, we showed the importance of melanin for determining the fate of intracellular pH in a single apoptotic cell.

## Introduction

**Fig 1:**
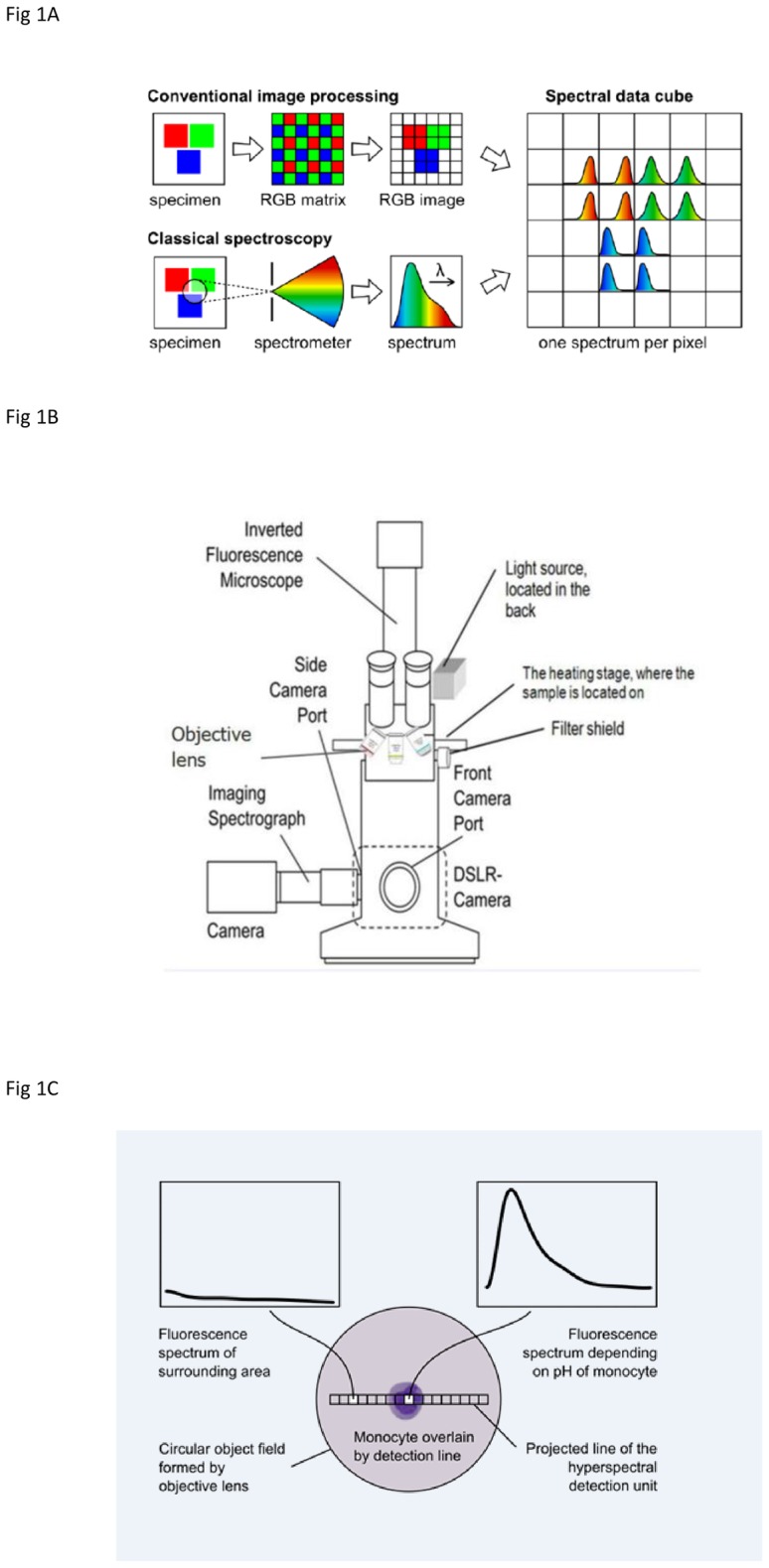
Principle of hyperspectral imaging (HSI) as a combination of conventional image processing and classical spectroscopy. **(A)** Hyperspectral data cube includes a set of data that are layered on top of one another. Each pixel in the cube consists of an entire spectrum and the resulting image represents the corresponding wavelength band. Typically in hyperspectral imagery, the spatial information is collected in the X-Y plane and spectral information represented in the Z-direction [44]. **(B)** HSI detector attached to side camera port of an inverted microscope. This combination comprises the imaging spectrograph with sensitive monochrome camera. The CCD camera attached to the spectrograph and the main body through a port. It records the spectral images in a time interval of microsecond-scale. The fluorescence light source is located in the back of camera segment and is adjustable to give an ample light intensity. The front DSLR camera obtains real images of the sample that is located on the heating stage. The filter shield contains combination of filters to detect different fluorescent probes with different excitation/emission rate. **(C)** Different spectral responses at distinct positions within the detection line. The monocyte is placed within the object field. The multiple fluorescence spectra of cell, conidium or their surroundings are simultaneously captured. The schematic shows a line of areas, and each line covers more than one pixel.

The Hyperspectral imaging (HSI) technique is based on the combination of classical spectroscopy and conventional digital image processing [1]. In contrast to the conventional imaging technique or confocal fluorescence microscopy; in which the wavelength selectivity is obtained by positioning band-pass filters within the optical path [2], HSI collects a complete fluorescence spectrum from each point of measurement [3]. HSI records spectral and spatial information of a sample and processes a tremendous amount of data at the same time [4]. Whereas the color of visible light in three bands (red, green and blue) are detectable by human eyes, spectral imaging system can divide the light spectrum into many more bands. In the spectrometer through an optical dispersing element such as grating or prism, the light is split into various narrow bands and the information of each band is stored into the data cubes [3] as shown in the illustration (Fig 1A). Although the general RGB color (or multispectral) imaging techniques can be more cost-effective [5], HSI as a complete system including the camera and software appears to be more sensitive, specifically when it comes to the real-time measurements [4]. The prefix “hyper” stands for the large number of wavelength bands that fluorescent dyes are discriminated through them [6]. Typically, these numbers are much greater than the 3 bands of RGB cameras [7] whereas in our study they are 35 with covering more than 200 data points in the fluorescence spectra. Ignoring the real spectral resolution of the imaging spectrograph, this is a nominal value that specifies the resolution of the spectrograph (here is 7 nm).

There are several scanning methods to acquire hyperspectral data cubes through comprising two spatial axes and one additional spectral axis.

The so-called whisk broom scanner collects HSI data by point scanning [8] and spectral laser scanning microscopes work based on this method. They are supplied with one-channel-spectrometer that scans the sample in two dimensions.

Another subtype of HSI is the push broom scanner, which performs the line scanning. The application is based on measuring a series of points that form a line with the sample and is scanned in one dimension. This method is also applicable for the linear measurements when there is no need for spatial scans [9].

Another optical system for capturing HS images uses wavelength scanning, i.e. in tunable filters [10] or FTIR [11].

Furthermore, there are full data cube snapshot imagers with no requirement for scanning the image [12]. Such novel devices are just becoming commercially available and gradually being equipped with larger arrays of detector elements [13].

The scanner that we used in our devise is a 4-color-microarray pushbroom. However, as the measured samples were not motile, there was no use for the scanner but for a spectral imager. Then from each part of sample, series of image were constantly acquired and analyzed.

Nowadays, the use of imaging spectrometers has been highly increased due to easy access to the two-dimensional charge-coupled device (CCD) (which also exists in our setup) and complementary metal-oxide semiconductor (CMOS) arrays. During the first decade of emerging SI technology, the choice for the most sensitive detectors was limited. Formerly, only CCDs were known as the highest performance array detectors but ever since, CMOS as the low-noise sensors have been widely applied [14]. Currently, CCD and CMOS sum the multiple snapshots in different ways. CMOS mostly combine voltage signal whereas CCDs combine signal charges [15]. Nevertheless, both of these imagers convert the light into electronic signals and deflect the light radiance from each pixel of an image. They produce various extended spectral bands and therefore, each pixel in the image contains the entire spectrum, thus a highly resolved image with detailed information is provided [16]. Along with the wide expansion of HSI application as an assessing non-invasive technique in food industry during last decades [17], recently it has been applied in various fields of biology i.e. to determine disorders in plants caused by bacteria or fungi [18, 19, 20], to categorize the microbial populations [21], to identify the pathogens in clinical microbiology [22] and profiling the tumour cells [23].

In comparison to classical fluorescence microscopy, HSI greatly rectifies the measurement process, since there is not any need for using narrow band filters and also no advanced knowledge is required for selecting the emission filter to match the dyes. Post-processing the recorded spectra gives all information from the data set. Therefore, when an experiment is conducted based on measuring the mixed fluorescence signals from different components or compartments, HSI would be the suitable method to generate the transmitted signals and corresponding information from several sources within one single sample [24]. These samples can include a broad range of spectrally overlapping fluorescent labels on any microscopic slides, buffer solutions or cellular biomolecules. By analysing the spatial relationships between the various spectra in any neighbourhood, this method enables the user to elaborate spectral-spatial models for an accurate segmentation and classification of the image, i.e. within a cellular compartment or between some cells in a culture medium [25]. Also, measuring the spectral resolution is one of the most important features that this method provides. The spectral resolution determines the maximum number of spectral peaks that are resolved by spectrometer [26]. Additional advantage of HSI is provided by the massive capacity of data recording, combined with computer-based analytical methods. This asset potentially allows the subsequent separation of the constituent values i.e. discrimination of multiple overlapping fluorescent labels. This general feature represents a powerful tool to detect the distribution and contribution of chemical or biological compounds in the sample of interest. In biological assays, the capability of instantaneous real-time detections from different spots of a live sample in long term measurements emphasises it greatly among other microscopic systems like confocal microscopy. Last but not least, in single cell studies when the cell cycle and different stage of apoptosis are at the focus in-vivo, those cell populations that potentially contain a mixture of apoptotic and necrotic cells together, can not be accurately distinguished by the common standard techniques like Fluorescence-Activated Cell Sorting (FACS) [27].

In this study, we aimed to record the entire spectrum from each data point. Therefore a targeted analysis of defined cell fate and pH including different stages of apoptosis upon melanin infection was facilitated based on HSI method. Here, we describe the modification and application of a commercial fluorescence microscope, which we upgraded with HSI technology (Fig 1B). This construction enabled us to follow simultaneously the emission of different fluorescent dyes in single living cells upon infection with pathogens. In 2005, Roger Y. Tsien addressed the indicator molecules and proteins that are bounded to different cell compartments as “Spies” [28]. In our study, intracellular fluorescence spies allowed us to perform kinetic studies of some intracellular, infection-related events in parallel, like the measurement of pH or apoptosis (Fig 1C).

In recent years, many studies have been performed to understand the events during host cell- pathogen interactions [29, 30, 31]. Their results indicate that within the infected host cells, programmed death or apoptosis is one of the most common immune responses and it maintains the homeostatic balance [32]. So far, the events leading to the initialisation of apoptosis, its progress to different stages or its inhibition were broadly investigated and intracellular pH during apoptosis has been considered as a critical parameter for the cell’s function [32, 33]. Although during apoptosis, the cellular pH is dramatically changed [34], [35], its exact role during apoptosis remains unclear [36].

Among several microorganisms, the human-pathogenic fungus *Aspergillus fumigatus* is a prominent example, that can regulate apoptosis by preventing the release of cytochrome c and protect the mitochondria [37]. *A*. *fumigatus* is one of the most important air-borne fungal pathogens [38]. With the dihydroxynaphthalene (DHN) melanin pigment (present in wild-type spores or conidia), the fungus is capable of antagonizing the acidification of phagolysosomes in macrophages [39]. Although the majority of infectious particles induce apoptosis in host cells [40, 41], the melanised conidia of *A*. *fumigates* can manipulate the immune system by regulating apoptosis [41]. This could be explained by the fact that DHN melanin is a good electron acceptor and can capture the highly reactive free radicals which are generated during apoptosis [42]. *A*.*fumigatus* has shown the ability of inhibiting the extrinsic apoptotic pathways [41]. It is already known that when apoptosis occurs, the intracellular pH is acidified [43] but still yet to be determined whether an apoptotic cell is infected with *A*. *fumigatus*, how the cell’s destiny including pH would be upon infection. Here, by applying HSI which allows a comprehensive analysis at the single cell level, we report the detailed analysis of apoptotic human monocytes carrying pigmented *A*. *fumigatus* conidia.

## Material and Methods

### Cell cultures

Human monocyte cell line (MM6) [45] (obtained from DSMZ Braunschweig, 2007) in suspension were maintained in 11835 RPMI 1640 no phenol red Medium (Life technologies, Darmstadt, Germany) supplemented with 10% (v/v) heat-inactivated fatal calf serum (FCS) (Lonza, Verviers, Belgium), MEM non-essential amino acid 100× and 10 mg/ml gentamicin (PAA Laboratories GmbH, Cölbe, Germany). Incubations were carried out at 37°C in a humidified incubator with 5% (v/v) CO_2_.

### Fungal strains, culture condition and harvesting of conidia

*A*. *fumigatus* ATCC 46445 wild-type strain and the *pksP* melanin-free mutant derived from ATCC 46645 [46] were propagated on *Aspergillus* minimal medium (AMM) [47] agar plates at 37°C. On day 5, the conidia were harvested by rinsing the culture plates with 10 ml 0.9% (w/v) NaCl solution supplemented with 0.1% (v/v) Tween 20, and filtered through 40-μm cell strainer (BD, Heidelberg, Germany). A fresh conidia suspension was incubated in RPMI 1640 no phenol red, without FCS for 3 h at 37°C, and then washed twice with sterile phosphate-buffered saline (PBS) supplemented with 1% (v/v) Tween 20. Concentration of conidia was calculated by using a Thoma chamber and microscopic according to the classical method [48].

### Phagocytosis assay

To infect the cells, first conidia were labeled with Fluorescein isothiocyanate (FITC) (Sigma-Aldrich, Germany) [49] and counted. 2 × 10^6^ MM6 monocytic cells/well were seeded with conidia in the ratio of 1:1 up to 1:5. The plates were centrifuged for 10 min at 100× g, at 37°C to synchronize the conidial exposure event and the cell/conidia suspension was incubated at 37°C, 5% (v/v) CO_2_ for 6 h. Cells again were centrifuged at 900 × g for 5 min at 4°C and washed. To stop phagocytosis, the cells were incubated with ice-cold PBS for 30 min, followed by 3 times washing steps with PBS. Also as the control for apoptosis inhibition, prior to the infection, some samples were treated with 2 μM cytochalasin D (Sigma-Aldrich, Germany) for 1 h at 37°C [50]. After three times washing steps, the ingested/unbound conidia were detected using a fluorescence Olympus B×51 microscope (Olympus, Hamburg,Germany). From two independent experiments, three random fields containing around 100 monocytes per field of view were considered to calculate the rate of phagocytosis using the formula: number of phagocyted conidia/total number of conidia in the culture ×100.

### Apoptosis induction

To induce the mitochondria-mediated apoptotic pathway and initiate intrinsic apoptosis [51], Staurosporine (STS) (Life technologies, Darmstadt, Germany) was used. Cells were cultured in RPMI FCS-free medium and after 24 h of incubation cells were transferred to 6-well plates in the volume of 1 ml and 1×10^6^ cells. STS with the concentration of 1.5 μM was added to each well. After 4 h of incubation the cells were washed with PBS buffer once and resuspended in fresh media.

### Monitoring the apoptotic phases

To investigate the formation of apoptotic bodies and malformed cells, STS-induced cells were mounted on glass slides covered by cover slips and sealed with freshly opened nail polish. The changes in the nucleus, as well as cell shrinkage and deformities were observed under 1000 × magnification (Customized Nikon Diaphot TMD microscope). The progress of apoptosis within different phases from early apoptosis to necrosis was investigated by colouring the cells with annexinV conjugate Ex/Em 650/668 (Life technologies, Darmstadt, Germany). To label the cells with annexinV, the washed cells were resuspended in annexin-binding buffer {140 mM NaCl, 10 mM 4-(2-hydroxyethyl)-1-piperazineethanesulfonic acid (HEPES) (Thermo Fisher, Germany) (, 2.5 mM CaCl_2_, pH 7.4)}. 10 μl of annexinV conjugate was added to each well and it was incubated for 15 min at room temperature (RT). Thereafter, 10 μM of propidium iodide (PI): Ex/Em 533/617 nm (Thermo Fisher, Germany) was added to each well to assess apoptosis (AnnexinV^+^ PI^−^) and cell death (AnnexinV^+^ PI^+^). Cells were washed and injected into the micro channel and the signal variation from each apoptosis stage was detected hyperspectrally.

### Monitoring the cytosolic pH in apoptotic cells

Apoptotic cells were resuspended in Live cell imaging solution (LCIS) (Life technologies, Darmstadt, Geramny). 10 μl of pHrodo^™^ Green AM probe Ex/Em 509/533 (Life technologies, Darmstadt) was added to 100 μl Powerload^™^ concentrate (Life technology, Darmstadt, Geramny) and the resulting mixture was diluted into 10 ml of LCIS. The cells were centrifuged, washed and added to the mixture probe. After 30 min of incubation at 37°C, the cells were washed again with LCIS and injected into the micro channels to be measured hyperspectrally every 15 min. The pH calibration was accomplished based on the calibration kit provided by the manufacturer (Life technologies, Darmstadt, Germany).

### Co-localizing the pH sensitive probes

As an alternative way to detect the pH, instead of using labelled conidia, two different fluorescent pH sensitive beads where used: pHrodo^®^ Red Zymosan BioParticles^®^ Conjugate Ex/ Em 560/585 (Life technologies, Darmstadt, Germany), and also pHrodo^®^ Green Zymosan BioParticles^®^ Conjugate Ex/Em 509/533 (Life technologies, Darmstadt, Germany). 25 μg of the particles were added to 20 ml RPMI, 3.75 ml polyvinylpyrrolidone (PVP) solution (Roth, Karlsruhe, Germany), 750 μl water free Dimethyl sulfoxide (DMSO anhydrous) (Sigma-Aldrich, Germany), shaking at -8°C for 30 min followed by sonication for 5 min. The solution was replaced with fresh medium and added to the 1 × 10^6^ cells/well, including the experimental and no-cell background subtraction wells.

### Preparing multi-coloured cells

After 24 h of incubation in FCS-free medium, cells were washed and the nuclei were stained with 1 μg/ml DAPI (Sigma-Aldrich, Germany) in PBS for 15 min at RT. After apoptosis induction and 4 h further incubation, cells were washed once with PBS and coloured with annexinV. The cells were finally coloured with pHrodo^™^ Green AM conjugate.

### Labelling conidia with pH indicator probes

Freshly harvested conidia were washed with PBS/1% (v/v) Tween 20. For a better conjugation with dyes, the conidia were resuspended in the buffer solution pH 6.00±0.02 (Roth, Karlsruhe, Germany). To generate red-labelled conidia, a 1 mg vial of pHrodo^™^ Red succinimidyl (NHS) ester, MW = ~650 (Life technologies, Darmstadt, Germany) was added to 150 μl DMSO (10 mM dye concentration) + 0.1 M sodium bicarbonate buffer (pH 8.3) to the concentration of 10.2 mg/ml as stock solution. It was immediately disolved in DMSO resulting 1 mM of working solution. 1×10^8^ conidia were added to the working solution and after 40 min light-protected incubation at RT, the conidia were first washed with 10× Hank’s balanced salt solution, no phenol red (HBSS) (Life technologies, Darmstadt, Germany). Next, they were washed once with 1:10 diluted DMSO, and then with HBSS as the last washing step. To prepare green-labelled conidia, a 1mg vial of amine-reactive pHrodo^™^ green STP ester dye MW = ~750 (Life technologies, Darmstadt, Germany) was resuspended in 150 μl DMSO as stock solution. 0.1 M sodium bicarbonate buffer was added to adjust the pH to 8.3, and then it was diluted in DMSO to the final concentration of 1 mM as working solution. 1×10^8^ conidia were immediately added to the solution and the incubation time was optimised to 60 min at RT. The remaining steps were carried out similarly as described above. The pH calibration was accomplished based on the manufacturer’s protocol (Life technologies, Darmstad, Germany).

### Hyperspectral measurement of the intracellular pH in apoptotic multicoloured cells infected by labelled *A*. *fumigatus* conidia

Around 5000 apoptotic multicoloured cells were infected with labelled wild-type or labelled *pksP* mutant conidia. For ×1000 magnification, the compatible immersion oil (Immersol 518 F), (Zeiss, Germany) was used. Cells were injected into the micro channel μ-Slide Vl ^0.4^ (ibidi, Germany) and placed on the heating stage (Minitüb GmbH, Tiefenbach, Germany) of the hyperspectral microscope. Over the course of 35 h in intervals of 15 min, the emitted signals from each fluorescent dye were measured using corresponding filters. Meanwhile the images were taken in time intervals of 15 min by a camera attached to the microscope (Fig 1B). Each experiment has been repeated three times and at least two random fields containing approximately five macrophages per field were measured.

### Identifying the anti-apoptotic properties of melanin

To specify the function of melanin during apoptosis, we examined the effects of phagolysosomal pH modulator [52]. First, 1×10^5^ monocytes /well were induced by STS and incubated in the presence of 20μM chloroquine (Sigma-Aldrich, Germany) for 4 h at 37°C. After washing the cells with PBS, they were seeded with either wild-type or *pksP* labelled conidia.

### Examining the effects of melanin infection during phagolysosomal fusion

To evaluate the function of melanin during phagosomes and lysosome fusion, bafilomycin A1 was applied to secure phagolysosome merging and stimulate the acidification [53]. 1×10^5^ monocytes /well were induced by STS, treated with 100 nM bafilomycin A1 (Sigma-Aldrich, Germany) and incubated at 37°C. The cells were washed with PBS, and infected with either wild-type or *pksP* labelled conidia.

### Intracellular pH and standard curves

To generate the standard curves for the pHrodo red dye, freshly harvested conidia were suspended in 4 vials of 1ml pH buffer from pH 4 to 7, added with 1mM pHrodo^™^ Red AM intracellular pH indicator (Life technologies, Darmstadt, Germany), incubated in the dark for 40 min at RT. Conidia were sequentially washed with HBSS, then with 1:10 diluted DMSO and again with HBSS to effectively eliminate the unbound dye residue. Thereafter, conidia were transferred to different micro channels. For each pH value, the signals from 5 different conidia were hyperspectrally measured. To plot the standard curves, the average values from each 5 measurements were calculated and graphed (S1 Fig). To generate the standard curves for pHrodo green fluorescent dye, the cell-loading solution was prepared based on pH Calibration Buffer Kit (Life technologies, Darmstadt, Germany). MM6 cells in the concentration of 1x10^6^ were incubated with 10 mM pHrodo^™^ Green AM dye and 100 μl powerload^™^ concentrate for 30 min at 37°C. Cells were washed 3 times with LCIS (Life technologies, Darmstadt) which afterwards was replaced by cell-loading solution and incubated for 5 min at 37°C. These steps were performed for each pH buffer (pH 4–7) (S2 Fig). The relative signals for each pH value were plotted trough an average of 4 data points.

### Line detection

The pH-dependent fluorescence signal of monocytes was measured with a hyperspectral imaging detector and a commercial fluorescence microscope (Fig 1B). By using this combination, only a line within the conventional field of view was produced (Fig 1C). The device places a virtual cut through the cell of interest including its neighbourhood and the fluorescence profile is detected along this line. Thus, a one-dimensional image from the analyte is generated that its pixels possess the entire spectral information. The line detection method allows the sample to be observed at high repetition rates without any need for scanning since several hundred spectra are acquired simultaneously.

### Hyperspectral imaging (HSI) unit attached to HSI microscope

The HSI unit for the visible range was used. This unit is composed of an imaging spectrograph and a camera with the spectral range of 400–1000 nm. The spectrograph Specim V10E is assembled with 80 μm-wide entrance slit and provides the spectral resolution of 7 nm. The scale of its internal reproduction is 1:1 with 50% light transmission efficiency. It is connected to the camera and objective lens with the aid of two C-mount interfaces. The camera is a PCO Sensicam 680KU, supplied with a highly sensitive Electron Multiplying Charge Coupled Device (EMCCD) sensor for the low light settings. It comprises 1004 × 1002 pixels, binned to 1004 × 501 pixels and is cooled down up to 11°C. The HSI unit is operated by *pco*.*camwar*; proprietary software of the manufacturer (Kelheim, Germany). For spectral sampling, broadband color filters with different spectral profiles with an adjustable optical pass-band are located in the filter shield. The HSI unit is attached to the side camera port of the inverse fluorescence microscope Nikon Diaphot TMD without any magnifying or relay optics but instead, a custom-built C-mount adapter (Fig 1B). Half an hour prior to the measurements, the camera and light source turned on to stabilized and were kept in operation mode. For the spectral measurement of monocytes and conidia, a 40× microscopic lens (Nikon 40 DIC 0.55 LWD 160/0-2) was used. Considering the magnification, the HSI unit observes a thin line of 200 × 2 μm^2^ at sample level within the circular object field of 450 μm. Within the line five binned camera pixels acquire fluorescence along every 1 μm of the sample. The resulting spatial resolution of 0.2 μm per pixel is only a nominal value and is not provided by the optical system.

### Fluorescence excitation and detection

The fluorescence is excited by a Nikon epi-fluorescence attachment TMD-EF with a mercury arc lamp. Different interference filter sets were used to split excitation and emission channels. The filter cassette DM510 (excitation EX450-490, emission BA520) has been used for pHrodo Green and DAPI. For detection, pHrodo Red DM580 (excitation EX510-560, emission BA590) was used. AnnexinV was detected by using a Chroma filter cassette #49913 (beam splitter ZT640rdc, excitation ZET635/20×, emission ET655lp), (Chroma technology, Olching, Germany).

### Sample chamber

During measurement the samples were stored in a microchannel flow chamber μSlide Vl ^0.4^, exhibiting a physical surface modification that provides improved cell adhesion. The temperature of the chamber was controlled by a heating unit adjusted to 25°C. Higher temperatures were avoided to prevent excessive vaporization of the media. Microscopic images were taken using a digital full-frame SLR camera (Nikon D600) attached to the front camera port of the microscope.

### Spectral images

When an almost one-dimensional line was observed at the single cell level, the camera at the HSI unit acquired a two-dimensional image that detects light exclusively from this area (Fig 1C). The image showed an intensity distribution containing the fluorescence emission resolved in space (vertically) and wavelength (horizontally). By selecting a region of interest within the image, we could obtain the emitted spectrum of any part of cell or the background. To achieve the final image from the region of interest, three cell pixels were averaged and three background pixel were reduced.

### Image processing and spectral analysis

Monocytes and conidia were indicated by means of the fluorescence signal at the green and red region within the spectrum. Red fluorescence regions covered the spectral and spatial extent of the conidium. The monocytes were detected by the green fluorescence dye. To reduce the background signals, we smoothed the data using spline approximation. A spline is a piecewise polynomial function that is often used for modelling arbitrary functions like fluorescence spectra and was employed as a tool for fitting the polynomials to the original data [54]. Therefore, by fitting a spline to the original data, a smoother function was estimated while it simultaneously eliminates the noises. The calculation of the smoothed spectra was accomplished by the splev function from the SciPy collection [55]. SciPy is an open-source library for the programming language Python, created for scientific and technical computing reasons. Python is a broad advanced programming language with an easily readable syntax that can be extended by a huge number of function libraries for science and industry [56]. The SciPy function splev is based on routines of the library DIERCKX [54].

### Statistical analysis

When a single cell is considered as an in-vivo case study, it is unlikely to obtain exactly the same values from each measurement, nevertheless the pattern of intracellular changes should be identical [57]. With the HSI method, a plentitude of information from detailed structural and spectral data points can be collected. Yet, in this study, each experiment was repeated more than three times to ensure meaningful statistics. For statistical analysis, three data sets were plotted for each experiment (S4 Fig).To convert the plots, the standard plotting function of the base package of R programming language (version 3.2.3) was used (https://cran.r-project.org/src/base/R-3/). Each plot comprised of two y axis and one x. Followed by importing data from Excel to R, the second y axis showing pH values was plotted through the integral formula: 7–7.2*e-*4 * x + 9.35*e-*8 * x^2*–*6.39*e-*12 * x^. Then to show the time points, the x axis was plotted manually based on the recorded data. Finally to show the signal intensity the other y axis was plotted based on the original values generated by the microscope.

## Results

### Analysis of the impact of *A*. *fumigatus* conidia on monocytes

**Fig 2:**
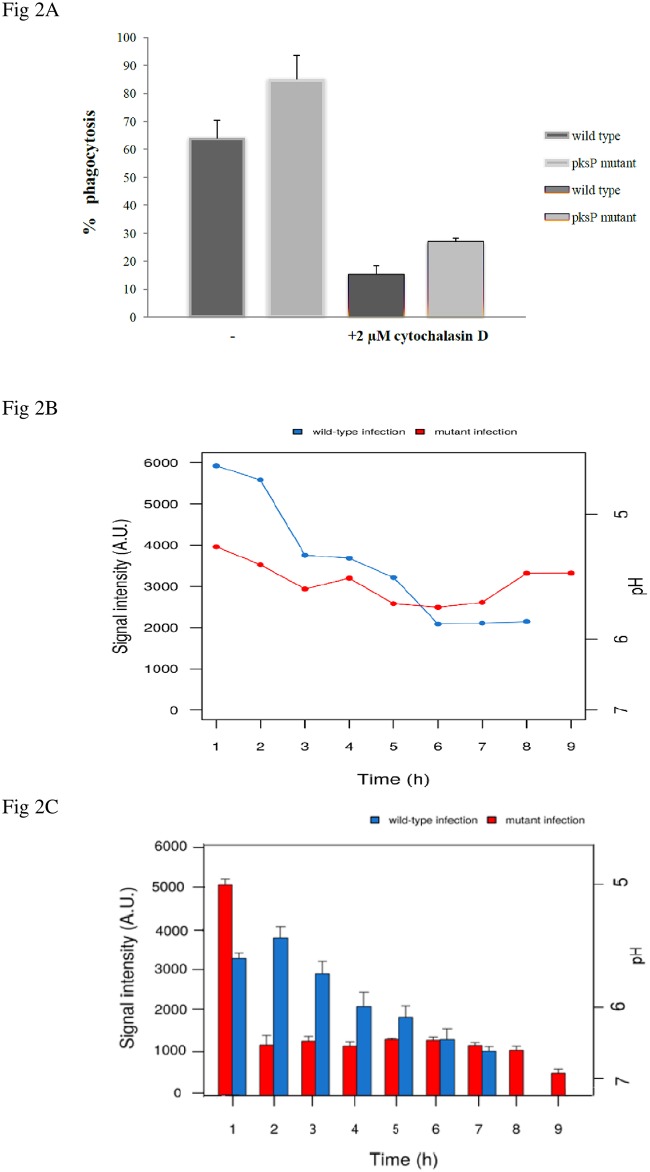
**(A) The effect of cytochalasin D treatment on phagocytosis of A.fumigatus conidia (wild-type and mutant)**. The cells that have been pretreated with 2μM cytochalasin D, showed a limited tendency towards phagocytosis the conidia in compared to the untreated cells. The 1 h treatment of MM6 monocytes with cytochalasin D reduced the rate of engulfed conidia to ~ 19% in wild type and 30% in mutant. **(B) pH variation in monocyte after infection with wild-type conidia (blue line) and the monocyte infected with *pksP* mutant conidia (red line)**. Intracellular pH was calculated via linear regression analysis based on fluorescence spectra ratio in comparison to the generated pH standard curve. In general, pH remained more acidic when the cell is infected with mutant conidia. **(C)** Data present the mean value _+_ SD of three experiments.

To determine how DHN-melanin influences human monocytes with respect to the pH of cells, we infected the cells with melanised wild-type conidia and with non-pigmented *pksP* mutant. As control, cytochalasin D was used prior to the infection (Fig 2A). The substance disrupts the polymerization of actin filaments that have a determining role during phagocytosis event. Hence, the cytochalasin D treatment would limit the phagocytosis consequently [58].

Once the conidia were bounded to the host, pH was measured over 9 h until the last signal was detectable (Fig 2B and 2C). After the successful phagocytosis, within 1 h the pH dropped to less than 5. Interestingly, 2 h post infection (p.i.) the pH started to recover gradually and reached nearly pH 6 after 7 h p.i. as a consequence of the dramatic cell shrinkage due to hyphae formation [59]. In contrast, when *pksP* mutant conidia were phagocytosed, pH constantly remained acidic compared to the wild-type conidia. After the first drop 1 h p.i. it slightly recovered to ~ 5.6 at 3 h p.i. and from this time onwards, it remained at 5.5 until 9 h p.i. when the first germlings were formed.

### Melanised *A*. *fumigatus* conidia recovered the intracellular acidic pH in apoptotic monocytes

**Fig 3:**
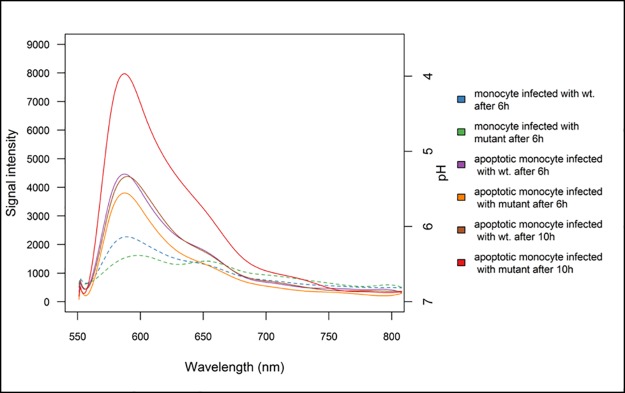
Spectrum of the pH at given time points (6 h and 10 h p.i.) in apoptotic and non-apoptotic infected monocytes. The dashed lines (shown in blue and green) are the spectra of pH in wild-type (wt.) and mutant infection in non-apoptotic monocyte at 6 h p.i. when their pH showed approximately similar values (6.3 to 6.5). The constant lines represent the wild-type or mutant infections in apoptotic monocyte (treated with STS) comparing two time points of 6 and 10 h p.i. The most acidic pH (less than 4) was recorded 10 h p.i. in the apoptotic monocyte infected with mutant whereas at the same time point the apoptotic monocyte infected with wild-type showed a higher pH around 5.3.Regardless of the type of infection, the signals related to acidic pH in non-apoptotic cells were generally less intense than in apoptotic cells. 10 h p.i., the apoptotic cells infected by melanin-free *pksP* mutant conidia were far more acidic compared to cells containing wild-type conidia.

To evaluate the results from other studies showing that melanised conidia interfere with the apoptotic pathways [39, 51], we measured the pH in apoptotic and non-apoptotic cells upon infection. 4 h after induction of apoptosis, cells were infected with wild-type or *pksP* mutant labelled conidia. Also the non-apoptotic cells were infected accordingly. The entire spectra from engulfed conidia representing pH, were recorded versus the emission wavelength (Fig 3).

**Fig 4:**
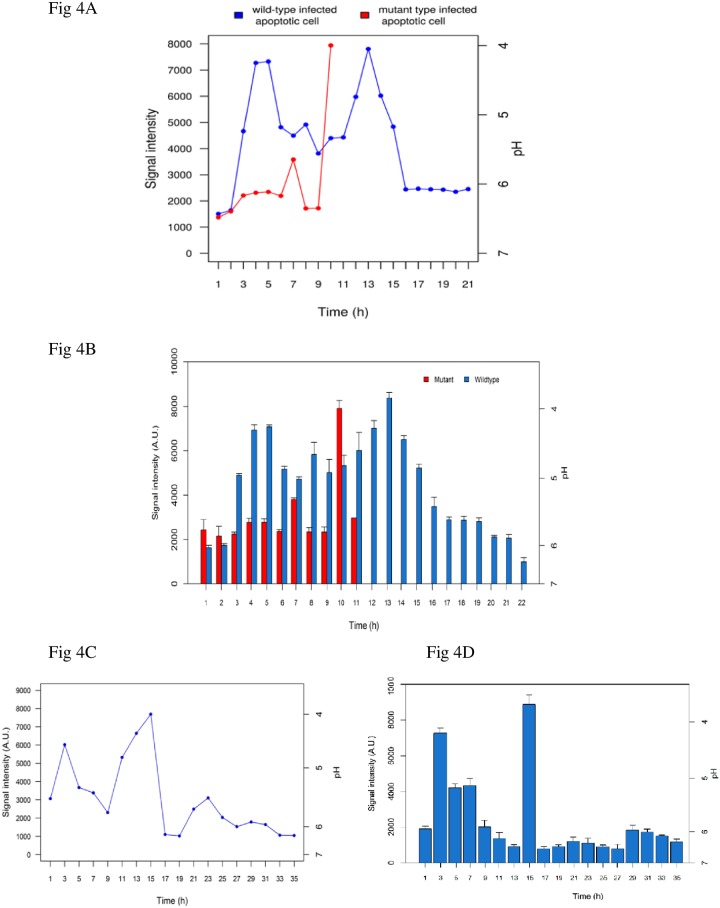
Kinetics of phagolysosomal pH upon infection. **(A)** Phagosomal pH in an apoptotic monocyte containing labelled wild-type conidia in comparison to an apoptotic monocyte carrying *pksP* mutant conidia. **(B)** Data represent the mean _+_ SD from three experiments. **(C)** Phagosomal pH in the survived apoptotic monocyte infected with labelled wild-type conidia. **(D)** Data represent the mean _+_ SD of cytosolic pH from three experiments.

Next, we demonstrated the effect of melanin on recovering the pH in apoptotic cells. An apoptotic monocyte was infected with labelled wild-type or mutant conidia. The constant measurement showed the gradual recovery from acidic pH towards neutral, whereas in an apoptotic monocyte carrying melanin-free *pksP* mutant conidia, the cytosolic pH did not recover (Fig 4A and 4B) and the host cell died as measured by propidium iodide staining (S6 Fig). By switching the corresponding filters, different fluorescent labels for each pH source were detectable. Since the mitochondrial-mediated apoptosis causes cytosol acidification [60], the measured pH was further investigated in the survived monocyte using filter cassette DM510 to detect green pHrodo. At time point 15 h p.i., the highest peak (representing the lowest cytosolic pH linked to the mitochondrial induced apoptosis) was observed (Fig 4C). It is conceivable that the fall of phagolysosomal pH at 15 h p.i., is related to the second peak observed at13 h p.i. from wild-type conidia in Fig 4A. Assumably, only after localization the melanised conidia in a mature phagolysosome the extreme acidic pH can be recovered. This finding demonstrates that in the presence of melanin, the cell could endure apoptosis that has been triggered through mitochondrial disintegration. Although at 15 h p.i. pH reached to 4, cell survived from the excessive acidic condition (Fig 4C and 4D).

### The effects of inhibition of phagolysosome acidification on the anti apoptotic properties of melanin

**Fig 5:**
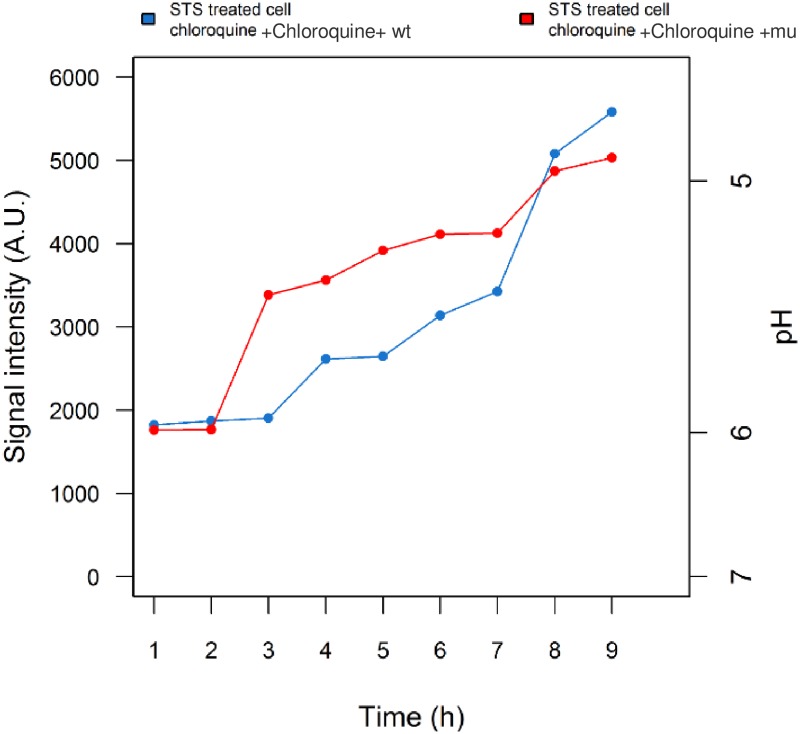
Effects of chloroquine treatment and phagosomal acidification on *A*.*fumigatus* infection in an apoptotic cell. The acidified pH of the apoptotic cell in the presence of chloroquine was maintained after infection. As it can be concluded, *A*.*fumigatus* only interferes with pH if the phagolysosme stays intact.

Following phagocytosis of conidia, phagosomes merge with endocytic organelles, i.e. the lysosome to form acidic compartments. These compartments are responsible for eliminating and digesting the pathogens [42]. It has been shown that melanised *A*.*fumigatus* can restrict the formation of phagolysosomes if it is successfully localised to the compartment [46]. Regarding the ability of chloroquine to stimulate phagosome and lysosome fusion [61], the acidified environment in the phagolysosome was maintained as it happened in the presence of *pksP* [51]. To examine the impact of melanin on the acidic environment in light of its anti-apoptotic properties, apoptotic cells were exposed to chloroquine. After stimulating apoptotic cells with chloroquine, neither wild-type nor *pksP* mutant infection reversed the pH or rescued the cells (Fig 5).

Chloroquine appears to be an inhibitor of caspases-3 that maintains the cellular pH [51]. As infected apoptotic cells did not recover from the acidic pH after chloroquine treatment, we concluded that localization of melanin within the phagolysosome is crucial for inhibiting apoptosis.

**Fig 6:**
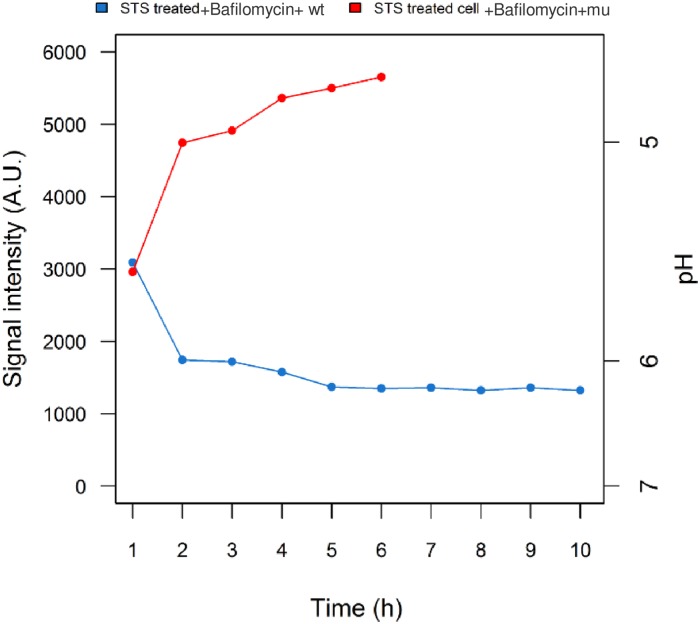
Effect of bafilomycin A1 treatment and inhibition of phagosomal acidification. Despite the inhibitory effect of bafilomycin on phagosomes-lysosome degradation, in the presence of melanin the engulfment of conidia and inhibition of intra-cellular acidification were not affected and the conidia were localised in the phagolysosome. Wild-type infection prevented acidification of phagosome, while the *pksP* mutant was not able to recover the acidic pH.

To distinguish the alternative possibilities that *A*.*fumigatus* governs the cell acidification inhibition during apoptosis, the further degradation of phagosome-lysosome as well as phagolysosome acidification was perturbed using bafilomycin. Bafilomycin is a chemical agent that selectively inhibits the vascuolar ATPase (vATPase) and keeps phagolysosom from further maturation [62]. Interestingly, in the presence of bafilomycin in the nanomolar range, melanised *A*.*fumigatus* completely inhibited intra-cellular acidification and cells endured apoptosis, whereas infection with *pksP* mutant did not recover the pH (Fig 6). These findings are in agreement with previous work showing that vATPase is a possible target for wild-type *A*.*fumigatus* to inhibit the acidification [39] and modulate the fate of apoptosis as a result.

## Discussion

In apoptosis-related assays, if the cell populations potentially contain necrotic, apoptotic and viable cells together at the same time, the final result will not be reliable. Since in the standard bulk techniques i.e. FACS, western blot or DNA electrophoresis, distinguishing between early and late stage of apoptosis is not possible, performing various types of assays are required for an accurate result. However such assays always need large amount of cells, therefore the experiments in the level of single cell are excluded. In addition, fluorescence staining methods using conjugating antibodies; which are dependent on specific binding between apoptotic-related probes and antibodies, are not suitable for the real-time monitoring. Whereas all these methods are based on invasive approaches leading to the cell membrane permeabilization [60]. The alternative technique that is commonly used to detect the fluorescence probes is confocal microscopy [63]. Nevertheless, the generated background signals while measuring the labeled biospecimens are variable in confocal microscopy and this might cause the blurred images and consequently an inaccurate result. Also to determine the different stages of apoptosis, additional complementary assays i.e. caspases tracking are required [60,63]. Regardless of multi-color or monochrome labeling, the quality of images in confocal microscopy is determined by spatial resolution. Then during long-term measurements, if under any circumstances the image becomes blurred, the data might be misinterpreted. Since in this study, we aimed to record the entire spectra from several data points in one sample, HSI was the well-suited tool to the outlines of the project.

Monocytes are motile phagocytic cells of the innate immune system, that are involved in the intracellular killing of microorganisms [64]. Throughout all host inflammatory responses; including apoptosis, the number as well as the function of monocytes are regulated [39]. Thus, determining the surviving monocytes during apoptosis, could be an adequate way to investigate the pathology of different diseases and the potential strategies for their treatments. Regardless of the origin of pro-apoptotic caspases, in both mitochondria and death receptor-mediated apoptosis [34], pH of the cytosol drops. Also, it has been demonstrated that melanised *A*. *fumigatus* conidia can prevent the acidification of phagolysosomes to a certain extend [39] and regulate apoptosis as a result [51]. In this study we investigated the impact of melanin on the pH value of a single apoptotic monocyte and searched for a possible connection between cytosol acidification and intracellular processing of *A*. *fumigatus* conidia. To uncover the role of conidia uptake on pH changes, we used labelled *A*. *fumigatus* conidia as intracellular spies of pH in apoptotic host cells. Also, by applying HSI technology, we recorded the kinetics of intracellular events in single cells.

Since cells are usually alive and show no shrinkage during the first 4 h after STS treatment [41], this time point was chosen to co-incubate the apoptotic cells with melanised wild-type and non-melanised *pksP* mutant conidia. By measuring the pH spectra in two individual apoptotic monocytes which had either been infected with melanised conidia or white *pksP* mutant conidia, we demonstrated the effects of melanin on recovering cellular pH. It is observed that, once an apoptotic monocyte has been infected with wild-type conidia, the apoptotic cell survives by gradually recovering from the acidic pH. In contrast, in an apoptotic cell infected with melanin-free *pksP* mutant conidia, the cytosolic pH does not recover. This suggests that in the absence of melanin, the mutant conidia do not interfere with the apoptotic cell fate. Consequently, cells remain in the acidic environment and enter the lethal phase. We related this difference to the presence of melanin, as it is a component that protects the fungi against the host immune system [65].

To verify whether the phagolysosome pH can be altered by melanin during apoptosis, two chemical substances were selected. Chloroquine and bafilomycin are both autophagy inhibitors that regulate the acidification of the phagolysosome [53]. Chloroquine is an anti-malaria drug and a weak base [66] that can partition acidic vesicles (i.e. phagosome and lysosome). It increases enzyme activities in these organelles and reduces the pH by increasing the reactive oxygen species [61, 67]. We observed that when an apoptotic monocyte is exposed to chloroquine, infection with either wild-type or melanin free *A*.*fumigatus* mutant maintained the acidic pH as it was imposed at first place by apoptosis. Taking into consideration the role of chloroquine in acidification of the phagosome [68] and the controversial effects of melanin after chloroquine treatment (Fig 5), we concluded that chloroquine has the ability to prevent the anti-acidic properties of melanin in the apoptotic cell and melanin can only reverse the acidic pH when it is located in the phagolysosome.

In parallel, the role of bafilomycin A1 in blocking lysosomal degradation, driving phagosome-lysosome fusion and inhibiting their proteolysis [69] upon melanin infection was examined. After induction of apoptosis, the lysosome degradation was further inhibited by bafilomycin A1 [39]. Nevertheless, the wild-type conidia were still competent to reverse the pH through internalisation to the phagosomes. The proposed effects of melanin were validated in the mutant infection, in which the acidic pH was preserved as expected (Fig 6). Since bafilomycin through the inhibition of vacuolar ATPase, prevents the phagosomes and lysosome merging independent of lysosomal pH [70], it is highly possible that *A*.*fumigatus* modulates the apoptosis through the restriction of vATPase.

Two peaks were observed the pH profile during infection with wild-type conidia. We interpreted the first peak as the result of engulfment of conidia by the host cell. When a microorganism is engulfed into the cell, usually a phagolysosome is formed and the pH of this compartment becomes acidic in order to help digest engulfed material [71]. The second peak was consistently observed in the same timeframe across all measurements which indicates the acidic cytosol, and correlates with ongoing apoptosis. To investigate the cause and to ensure that the induced apoptosis had progressed *via* mitochondria-dependent pathways during infection and consequent pH recovery [34, 47, 72], we applied an alternative pH-sensitive dye to label the induced-infected monocyte. This allowed us to record the changes in phagolysosomal pH based on mitochondria-induced acidification, as an indicator of apoptosis progression [73]. Interestingly, in parallel to the phagosomal pH changes measured via conidia engulfment, we observed changes in lysosomal pH with a similar pattern. In addition, the signals from the cytosol of monocytes, showed the second peak in the same pH trend that appeared approximately two hour later. This finding demonstrates that the further conidial digestion had influenced on the cytosolic pH that had been reached to 3.7 during the experiment, and that the cell remained apoptotic during the entire event (Fig 4C and 4D).

Along with the hyperspectral measurements of fluorescent signals, a series of visual images were taken from *A*. *fumigatus* wild-type and *pksP* mutant conidia-infected monocytes, STS-treated monocytes, and the apoptotic monocytes infected with wild-type or *pksP* mutant conidia. Different apoptotic phases were determined from by different colour intensities, ranged from slightly reddish to the brightest red (S5 Fig). As time progressed, depending on the presence or lack of melanin, the fluorescent colour intensity changed, demonstrating the acidic pH recovery or pH dropping (S6B and S6E Fig).

In contrast to other fungi like *Candida albicans* which can induce its own uptake and initiate apoptosis [74], *A*. *fumigatus* conidia showed the ability to induce or modulate apoptosis in pulmonary epithelial cells [75] and alveolar macrophages [41]. The ability to prevent apoptosis from further progression appears to be imposed by obstructing mitochondrial acidification.

In summary, we have demonstrated how HSI; as a combined method that incorporates classical imaging technique and optical spectroscopy, can reveal spectral and spatial data of single cells. The method provides detailed information by recording the entire spectrum in each pixel of the whole image. We applied HSI to quantify the constituent pH variation in single infected apoptotic monocytes as a model system. *A*. *fumigatus* conidia can influence the host immune system [51] and interfere with the acidification of phagolysosomes [39]. Here, we extended this finding to monocytes and gained a more detailed view of the process. Our data indicate that melanised *A*. *fumigatus* conidia interfere with apoptosis in human monocytes by enabling apoptotic cell to recover from mitochondrial acidification and continue with the cell cycle. We also showed that this ability of *A*. *fumigatus* is dependent on the presence of melanin, since a non-pigmented mutant did not prevent the progression of apoptosis and consequently, the cell did not recover from the acidic pH. By conducting our research based on the HSI method, we were able to measure intracellular pH in single pro-apoptotic human monocytes and show the pattern of pH variation over 35 h of measurements. In conclusion, we have proven the importance of melanin for determining the fate of intracellular pH in single apoptotic cells.

## Supporting Information

S1 Dataset An example for pH calibration curves. Through the calibration of pH and the elimination of background signals, the standard curve for each pH range within the area of possible corresponding signals was determined. The calibration curves were used as the reference curves during analysis of the intracellular pH **(Text A). Statistical figures**: To provide the statistics, the experiments were repeated at least three times. Each figure includes three different data sets, plotted together **(Text B). Imaging different stages of apoptosis upon infection**: To complement our hyperspectral measurements of fluorescent signals, fluorescence microscopic images were taken. To determine the different apoptotic phases (early apoptosis, late apoptosis, necrosis and cell death phase), annexinV and propidium iodide (PI) were used to label the STS-induced cells and the cytosol was labelled with pHrodo green. Brighter fluorescence indicated the advanced apoptosis, while ultimately, the cells showed necrosis. by switching to the green filter, the early apoptotic cell could be distinguished (yield the lowest fluorescence) **(Text C). Comparing the images of apoptotic cells infected with wild-type and mutant conidia**: Within 4 h after induction by STS, the apoptotic but not necrotic cells were selected using PI and annexin V imaging and infected with *A*. *fumigatus* wild-type and *pksP* mutant conidia and the further changes in colour intensity were imaged. Although the monocytes infected with wild-type conidia (S6 A) were still apoptotic (S6 C), no acidic pH was detected around the conidia 7 h p.i. (S6 B), In contrast, apoptotic monocytes infected with mutant conidia (S6 D) under the same conditions showed acidic pH values (S6 E) as well as ongoing apoptosis (S6 F) **(Text D)**.
(XLSX)

S1 Fig Standard curves for pHrodo Red fluorescent dye. Each curve corresponds to a different pH value. The shift from each spectrum to the other is due to the change of pH. The lower pH causes the higher signal and at the neutral pH no signal was detectable (Text A in S1 Dataset).
(TIF)

S2 Fig Image of pH calibration in a single cell. The color intensity reflecting the pH 4–7. The pHrodo green pH sensitive fluorescent dye was used to label monocytes at different pH. The more acidic condition (D) caused the more intense color and in neutral pH (A) the monocyte is hardly seen.
(TIF)

S3 Fig Overview of the assay on hyperspectrally quantifying the intracellular pH in apoptotic cell upon infection. Signal from the region of interest, is recorded and separated from the background, then is converted to the graphable digits. Yet, more than one probe can be used and detected simultaneously sing corresponding filters.
(TIF)

S4 Fig Statistical plots. **(A)** The figures compare wild-type versus mutant infection in three data sets that are plotted together. The Fig 2B in the manuscript is the reference. **(B)** Statistical plot for the original Fig 4A in the manuscript. (**C)** Statistical plot for the Fig 4C in the manuscript (Text B in S1 Dataset).
(TIF)

S5 Fig Imaging different stages of apoptosis upon infection. Monocytes treated with STS and labelled with AnnexinV and PI. After 5 h of treatment and formation of apoptotic bodies, the necrotic cells were excluded from the assay. (A) No filter, (B) Chroma filter cassette 49913 (beam splitter ZT640rdc, excitation ZET635/20x, emission ET655lp), (C) Green filter cassette DM510 (excitation EX450-490, emission BA520) (Text C in S1 Dataset). l.a: late apoptosis; e.a: early apoptosis; n.: necrosis.
(TIF)

S6 Fig Images of apoptotic cells infected with wild-type and mutant conidia 7 h p.i. Apoptosis-induced monocyte infected with *A*. *fumigatus* wild-type and mutant conidia. (A, B, C) Images of labelled apoptotic monocytes were taken 7 h p.i. To track phagosomes acidification, cells were infected with wild-type conidia (labelled with pHrodo Red). To track the cytosol acidification, cytosol was labelled with pHrodo Green. After 7 h, when the pH had returned to neutral (A), the conidia within the cells were hardly detectable (B). The bright spots in the monocytes in image C, indicate that the apoptosis process is sustained. (D, E, F) Images of apoptotic monocytes infected with *pksP* mutant conidia. The higher intensity of red colour in image E shows the acidic condition in phagosome, contrary to the infection with wild-type conidia at the same time point in image B. The brightness of cell in image F indicates a strong acidic pH in the cytosol, resulting from mitochondrial-mediated apoptosis (compared to image C). (A, D): No filter, (B, E): Chroma filter cassette 49913 (beam splitter ZT640rdc, excitation ZET635/20x, emission ET655lp), (C, F): Green filter cassette DM510 (excitation EX450-490, emission BA520). Arrows point out the location of the detected cells (Text D in S1 Dataset).
(TIF)
