# Supplementary figures and images for: Hyperspectral Imaging Using Intracellular Spies: Quantitative Real-Time Measurement of Intracellular Parameters In Vivo during Interaction of the Pathogenic Fungus Aspergillus fumigatus with Human Monocytes
Source: PLoS One. 2016 Oct 11;11(10):e0163505. doi: 10.1371/journal.pone.0163505 (PMC5058474; doi:10.1371/journal.pone.0163505)

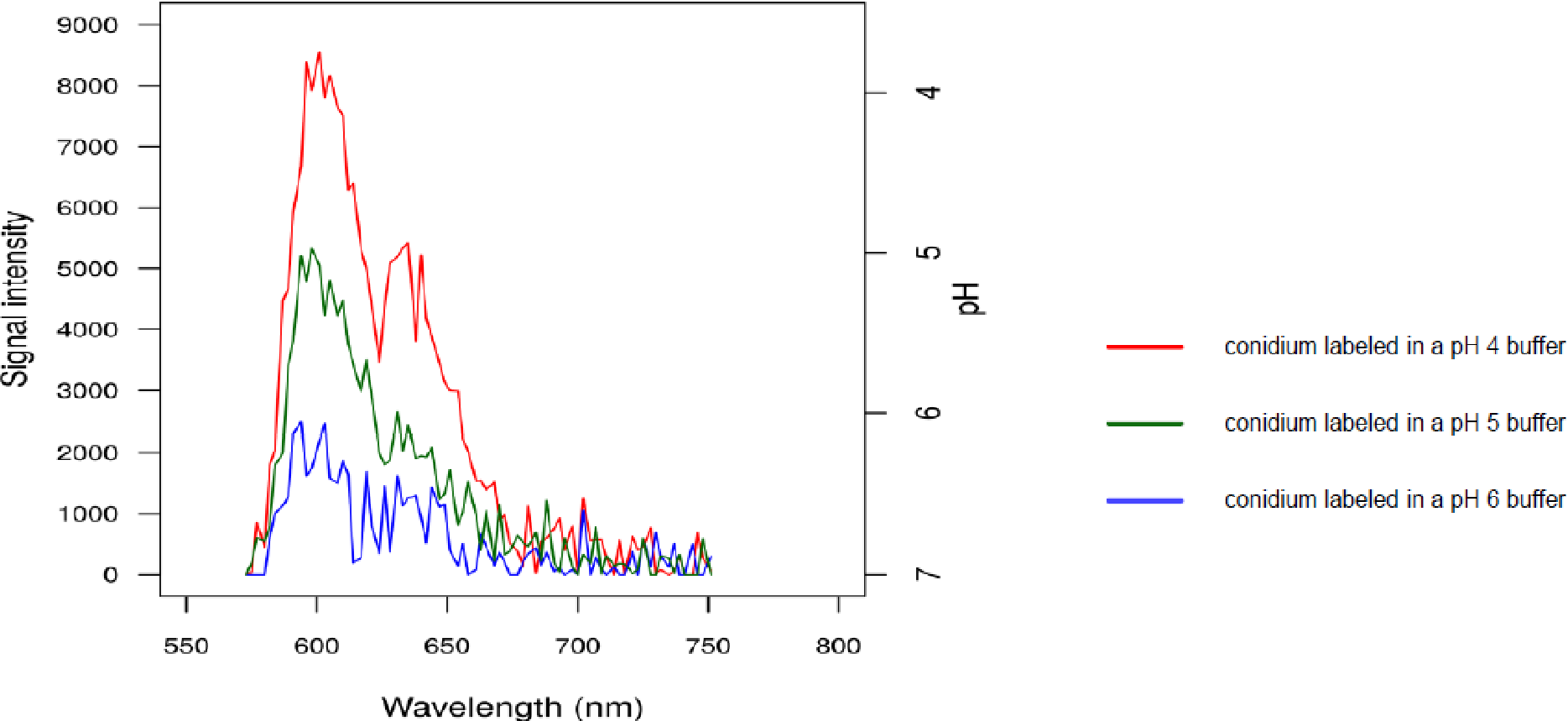

Supplement: S1 Fig — Each curve corresponds to a different pH value. The shift from each spectrum to the other is due to the change of pH. The lower pH causes the higher signal and at the neutral pH no signal was detectable (Text A in S1 Dataset). (TIF) [file pone.0163505.s002.tif]

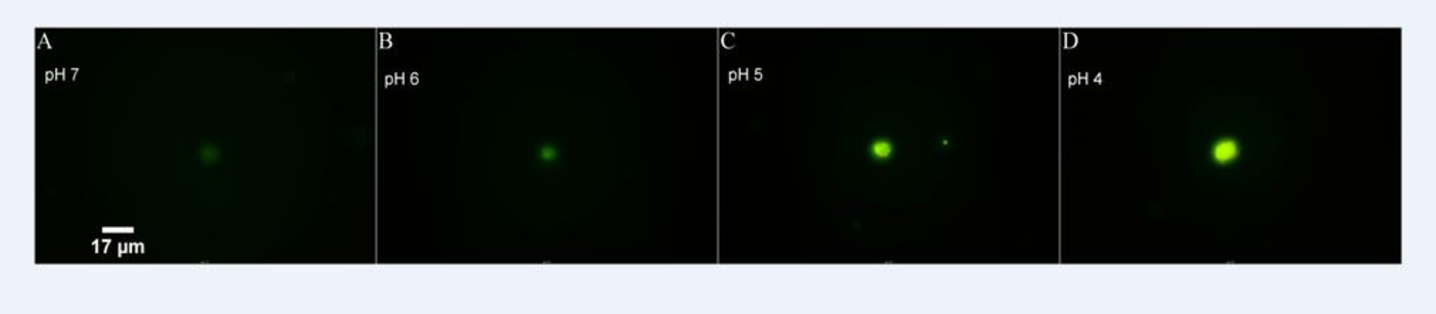

Supplement: S2 Fig — The color intensity reflecting the pH 4–7. The pHrodo green pH sensitive fluorescent dye was used to label monocytes at different pH. The more acidic condition (D) caused the more intense color and in neutral pH (A) the monocyte is hardly seen. (TIF) [file pone.0163505.s003.tif]

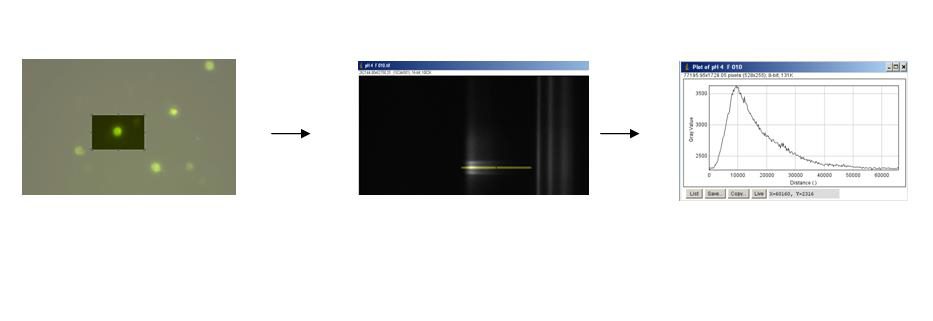

Supplement: S3 Fig — Signal from the region of interest, is recorded and separated from the background, then is converted to the graphable digits. Yet, more than one probe can be used and detected simultaneously sing corresponding filters. (TIF) [file pone.0163505.s004.tif]

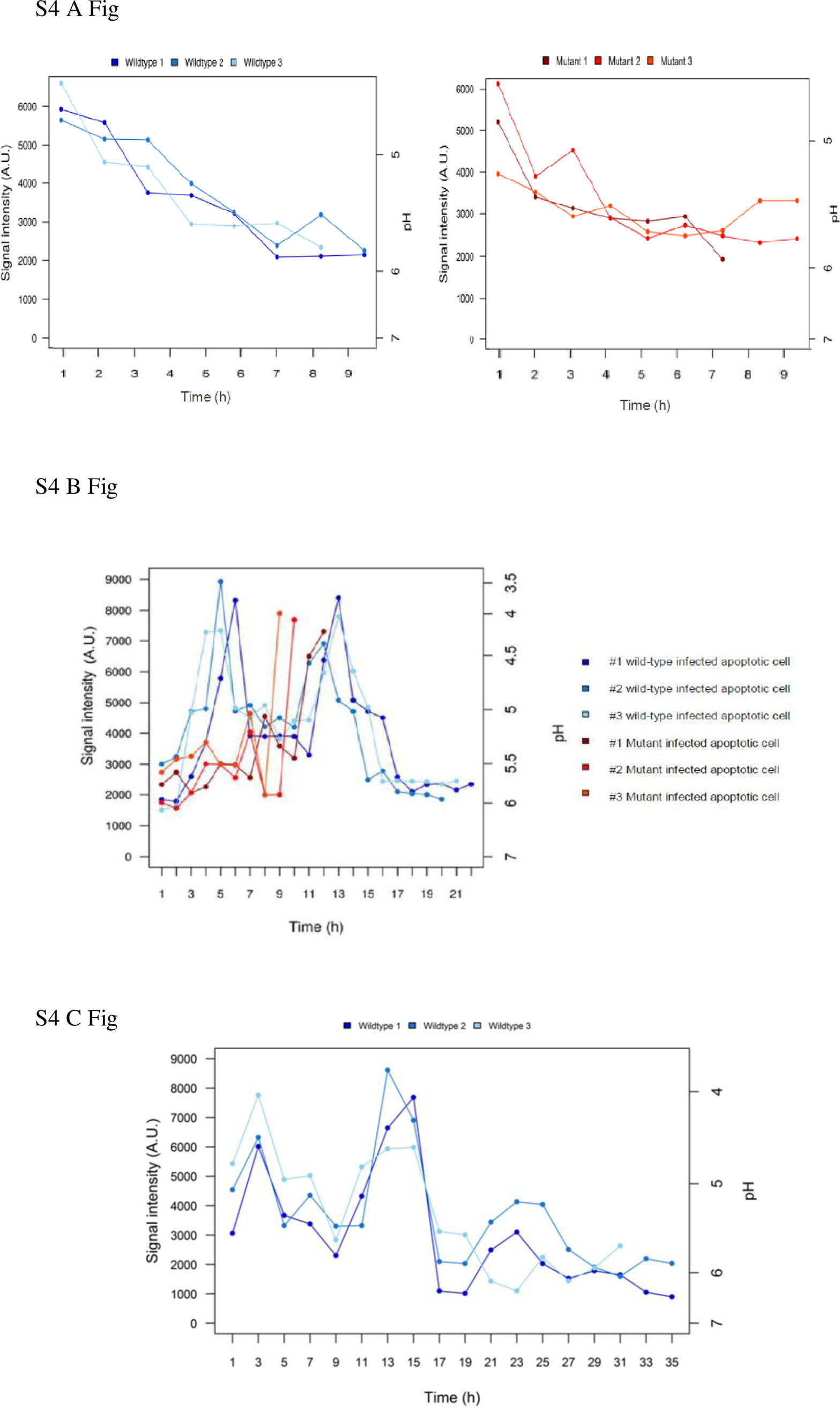

Supplement: S4 Fig — (A) The figures compare wild-type versus mutant infection in three data sets that are plotted together. The Fig 2B in the manuscript is the reference. (B) Statistical plot for the original Fig 4A in the manuscript. (C) Statistical plot for the Fig 4C in the manuscript (Text B in S1 Dataset). (TIF) [file pone.0163505.s005.tif]

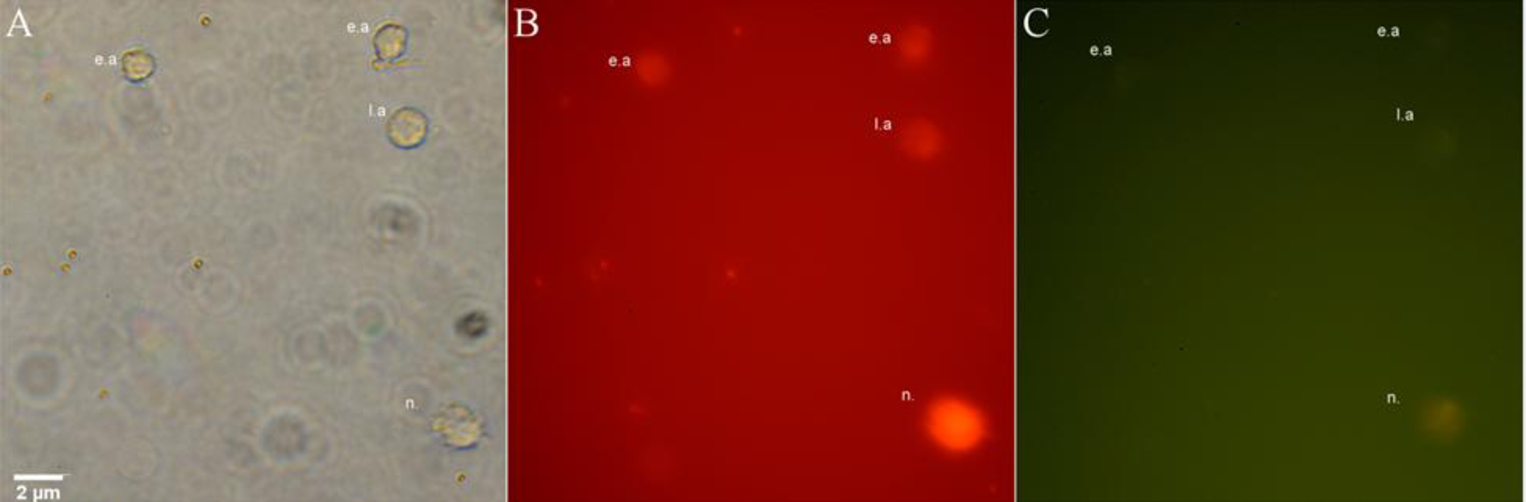

Supplement: S5 Fig — Monocytes treated with STS and labelled with AnnexinV and PI. After 5 h of treatment and formation of apoptotic bodies, the necrotic cells were excluded from the assay. (A) No filter, (B) Chroma filter cassette 49913 (beam splitter ZT640rdc, excitation ZET635/20x, emission ET655lp), (C) Green filter cassette DM510 (excitation EX450-490, emission BA520) (Text C in S1 Dataset). l.a: late apoptosis; e.a: early apoptosis; n.: necrosis. (TIF) [file pone.0163505.s006.tif]

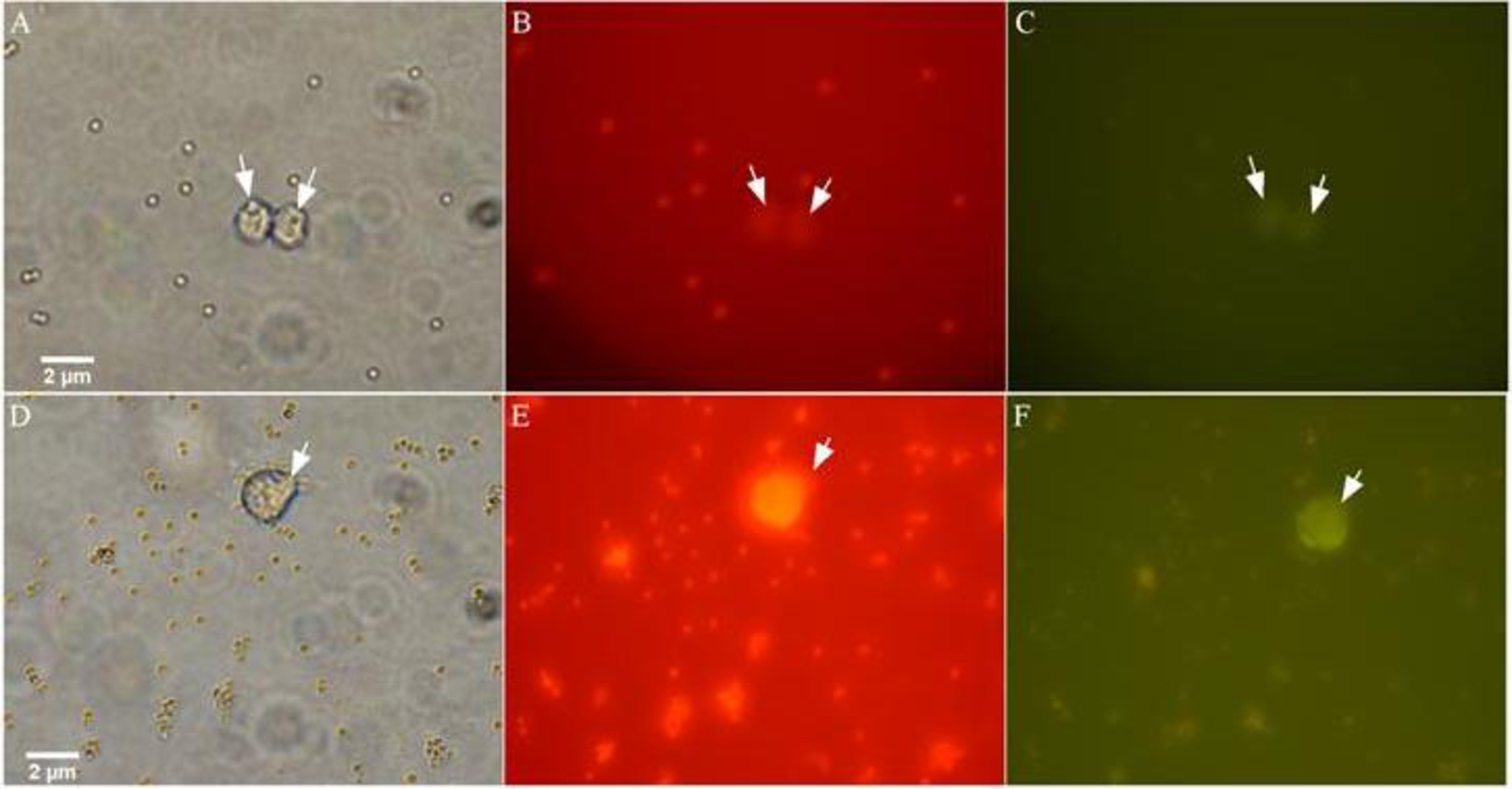

Supplement: S6 Fig — Apoptosis-induced monocyte infected with A. fumigatus wild-type and mutant conidia. (A, B, C) Images of labelled apoptotic monocytes were taken 7 h p.i. To track phagosomes acidification, cells were infected with wild-type conidia (labelled with pHrodo Red). To track the cytosol acidification, cytosol was labelled with pHrodo Green. After 7 h, when the pH had returned to neutral (A), the conidia within the cells were hardly detectable (B). The bright spots in the monocytes in image C, indicate that the apoptosis process is sustained. (D, E, F) Images of apoptotic monocytes infected with pksP mutant conidia. The higher intensity of red colour in image E shows the acidic condition in phagosome, contrary to the infection with wild-type conidia at the same time point in image B. The brightness of cell in image F indicates a strong acidic pH in the cytosol, resulting from mitochondrial-mediated apoptosis (compared to image C). (A, D): No filter, (B, E): Chroma filter cassette 49913 (beam splitter ZT640rdc, excitation ZET635/20x, emission ET655lp), (C, F): Green filter cassette DM510 (excitation EX450-490, emission BA520). Arrows point out the location of the detected cells (Text D in S1 Dataset). (TIF) [file pone.0163505.s007.tif]
